# Medium-Temperature-Oxidized GeO_*x*_ Resistive-Switching Random-Access Memory and Its Applicability in Processing-in-Memory Computing

**DOI:** 10.1186/s11671-022-03701-8

**Published:** 2022-07-05

**Authors:** Kannan Udaya Mohanan, Seongjae Cho, Byung-Gook Park

**Affiliations:** 1 Department of Electronic Engineering and College of IT Convergence Engineering, Gachon University, Seongnam-si, Gyeonggi-do 13120 Republic of Korea; 2 Department of Electrical and Computer Engineering with Inter-university Semiconductor Research Center (ISRC), Seoul National University, Seoul, 08826 Republic of Korea

**Keywords:** Medium-temperature oxidation, Germanium oxide, Resistive-switching random-access memory (ReRAM), Low-power hardware neural network, Processing-in-memory (PIM)

## Abstract

Processing-in-memory (PIM) is emerging as a new computing paradigm to replace the existing von Neumann computer architecture for data-intensive processing. For the higher end-user mobility, low-power operation capability is more increasingly required and components need to be renovated to make a way out of the conventional software-driven artificial intelligence. In this work, we investigate the hardware performances of PIM architecture that can be presumably constructed by resistive-switching random-access memory (ReRAM) synapse fabricated with a relatively larger thermal budget in the full Si processing compatibility. By introducing a medium-temperature oxidation in which the sputtered Ge atoms are oxidized at a relatively higher temperature compared with the ReRAM devices fabricated by physical vapor deposition at room temperature, higher device reliability has been acquired. Based on the empirically obtained device parameters, a PIM architecture has been conceived and a system-level evaluations have been performed in this work. Considerations include the cycle-to-cycle variation in the GeO_*x*_ ReRAM synapse, analog-to-digital converter resolution, synaptic array size, and interconnect latency for the system-level evaluation with the Canadian Institute for Advance Research-10 dataset. A fully Si processing-compatible and robust ReRAM synapse and its applicability for PIM are demonstrated.

**Graphical Abstract:**

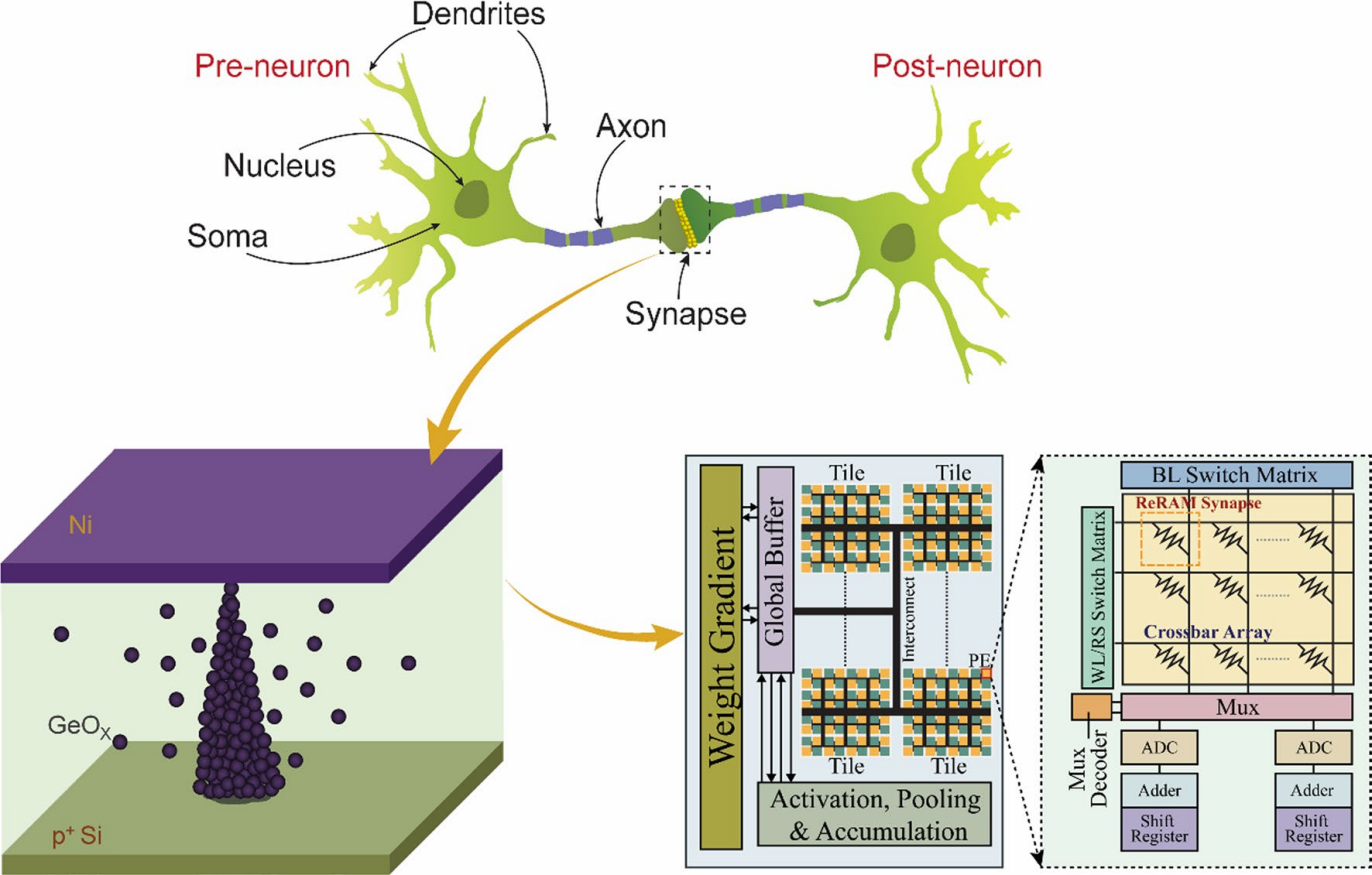

## Introduction

Over the past several decades, the physical downscaling in process technology is approaching the limits of fundamental physics. On the other hand, the demands on higher device scalability and operation speed, and low-power consumption capability have been incessantly increased, which gets more accelerated by necessity of data-intensive processing represented by big data analytics and deep learning for making accurate decisions in recent times. Conventional von Neumann architecture suffers from the memory bottleneck in this data-intensive applications due to the physically separated central processing unit and memory domain, along with the serial communication method between them. This inevitable serial data shuttling between the processing and memory domains leads to huge amount of latency and energy expenditure, which gets worse as the data size is required to be larger. Processing-in-memory (PIM) computing architecture has been researched for a long time in the very-large-scale integration (VLSI) technology regime for higher parallelism in data processing by introducing the processing capability into the memory domain [1–6]. However, most of the technological contributions have been made for the near-memory processing (NMP) in ways that the physical distance between processing and memory domains is reduced. The rather metaphorically used expression of PIM can be more substantially literal when supported by the device-level innovations. The PIM architecture design assures highly parallel computing capabilities which stem from the localized multiplication-and-accumulation (MAC) operations preferably using nonvolatile memories woven for the crossbar array toward higher area and energy efficiencies. Resistive-switching random-access memory (ReRAM) is considered as one of the most promising candidates for the synaptic components in the PIM architecture due to its simple device structure, high scalability, and fast switching speed [7–17]. Although researches on ReRAM devices have been focused on various aspects including device structure, electrode materials, and process integration based on wide variety of switching materials such as TiO_2_, NiO_2_, and TaO_*x*_, for higher device performances and reliability [18–20], there is still room for further improving the robustness of switching materials in terms of parameter distributions. For the qualification of ReRAM for the application as PIM component, higher device reliability should be warranted to endure the highly frequent learning and inference operations. Reliability of ReRAM devices has been a major concern, particularly in terms of distributions of low-resistance state (LRS) and high-resistance state (HRS) resistances with large deviations. Chou et al. fabricated Ni/GeO_*x*_/TiO_*y*_/TaN ReRAM device by room-temperature processing [21]. It shows an on/off ratio of 30 and a rather wide distribution of switching voltage. Cheng et al. reported Ni/GeO_*x*_/HfON/TaN ReRAM device with an on/off ratio of 900 fabricated at room temperature [22]. In a recent report, oxidation of Ge was pursued by an annealing at 600 °C, but the amount of GeO_*x*_ was small so that the HRS current was not effectively suppressed, which led to a small on/off ratio [23]. Also, it has been shown that GeO_*x*_ formed by an oxidation above 450 °C demonstrates an improved uniformity in surface roughness and the interface quality between the switching layer and electrode layers gets better [24].

In this work, electrically more robust and reliable ReRAM based on GeO_*x*_ as the switching material has been fabricated, characterized, and the system-level evaluations are carried out for the PIM architecture with the embedment of GeO_*x*_ ReRAM cells as the synaptic components. The switching layer of GeO_*x*_ was prepared by a medium-temperature oxidation (MTO) with a relatively higher thermal budget, in the opposite direction in which the ReRAM cells are usually fabricated by physical vapor deposition (PVD) at room temperature or at low temperatures not prominently higher than that. Based on the device operation parameters extracted from the measurement results, system-level evaluations of the PIM based on GeO_*x*_ ReRAM are performed with image recognition tests by series of simulations accommodating the realistic hardware circuitry. Detailed hardware performance parameters are presumed from a system-level simulation package for 32-nm technology node [25]. Last but not the least, the effects of nonideal ReRAM operation characteristic of variation in cycle-to-cycle operations on the hardware neural network performances are closely investigated.

## Results and Discussion

### Device Fabrication and Characterization

**Fig. 1:**
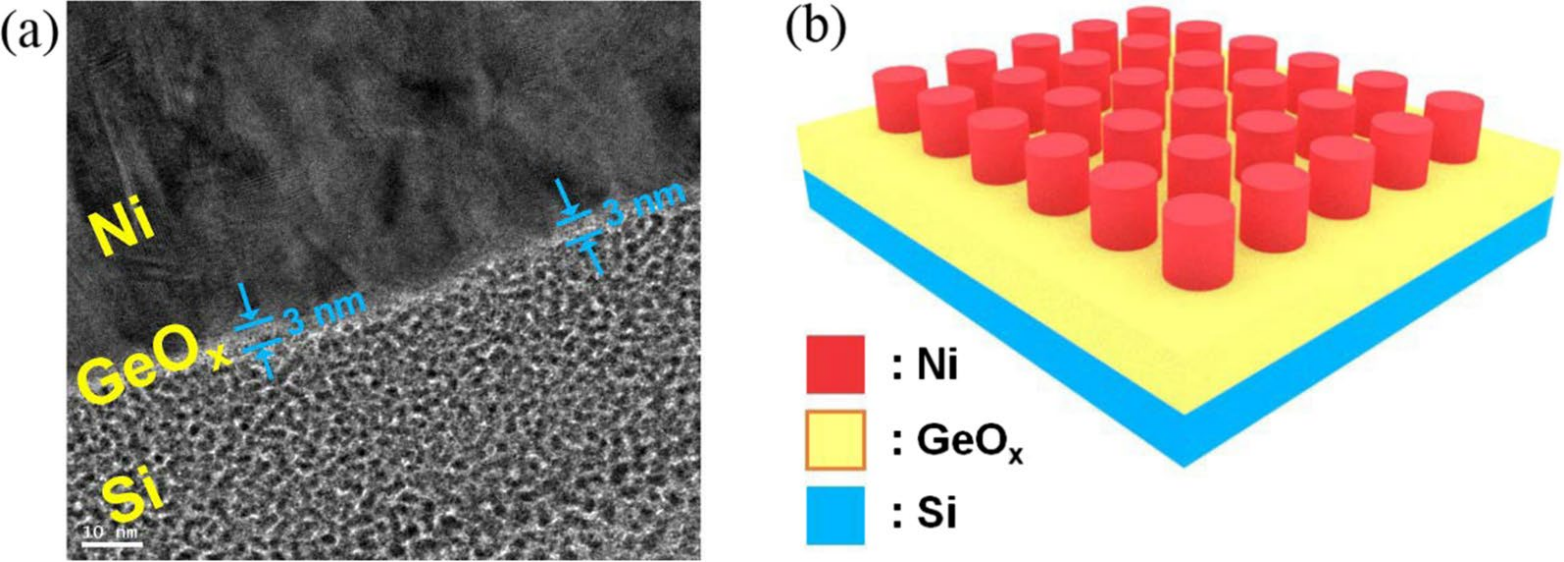
Fabricated GeO_*x*_ ReRAM cell. **a** Cross-sectional image by high-resolution transmission electron microscopy (HR-TEM). **b** Schematic of the ReRAM array in the Ni/GeO_*x*_/*p*^+^-Si metal–insulator–semiconductor (MIS) stack

**Fig. 2:**
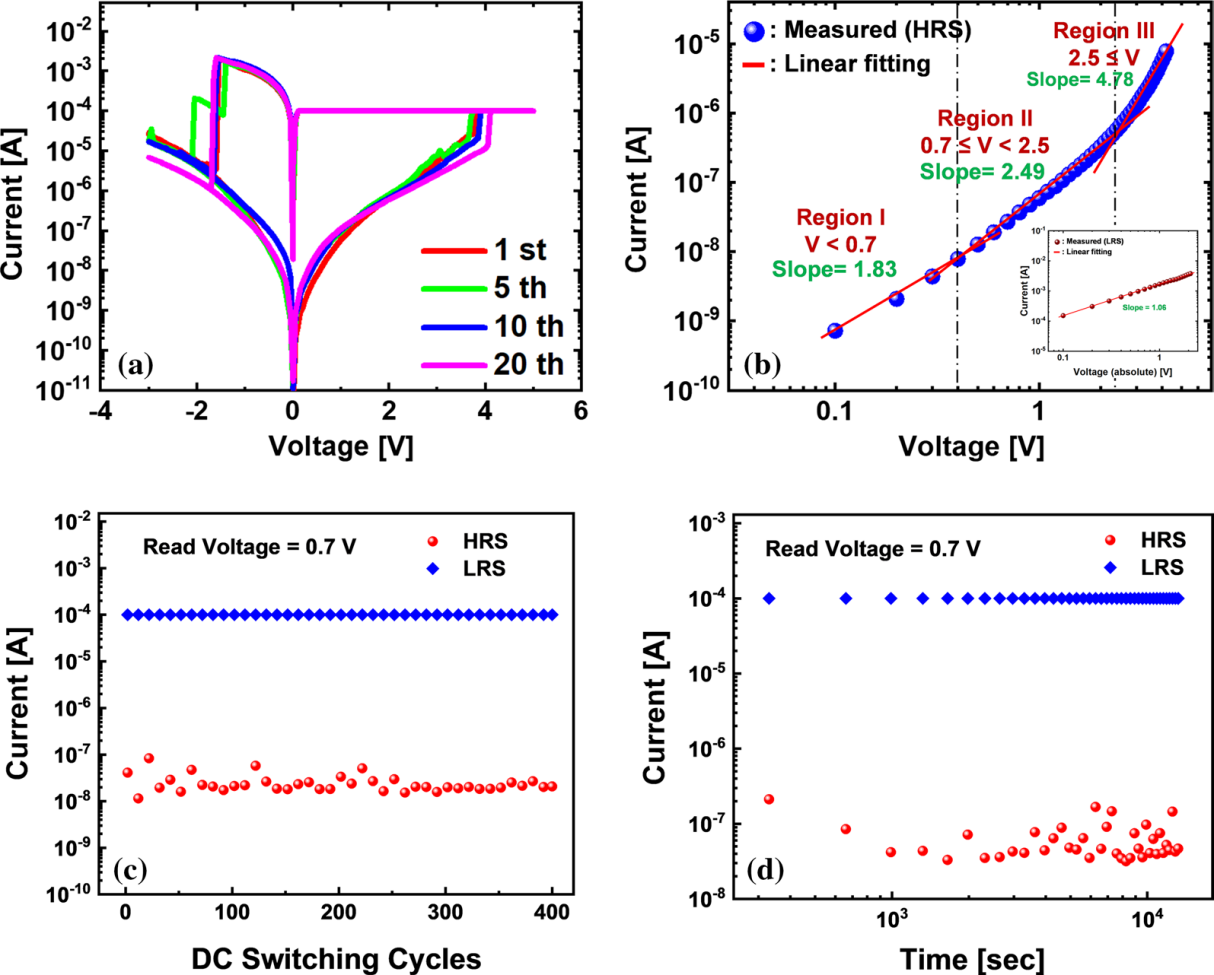
Measurement and fitting results from the fabricated GeO_*x*_ ReRAM cell. **a** *I*–*V* curves at 1, 5, 10, and 20 sweeps. **b** Double logarithmic *I*–*V* characteristics in the positive-voltage HRS region. Inset shows the negative-voltage LRS region. **c** Endurance and **d** retention characteristics of the fabricated ReRAM device

There have been various candidates for the material combination to make up the metal–insulator–semiconductor (MIS) stacks for ReRAM devices. In this work, Ni/GeO_*x*_/*p*^+^-Si MIS stack was fabricated. There are two reasons for having employed the material combination: one is to equip the fabrication viability through introducing the materials with compatibility to conventional Si processing which is mostly adopted for the modern VLSI electronics, and the other is to obtain more concentrated distribution of operation voltages with nonmetallic switching material. Figure 1a shows the cross-sectional view of a fabricated ReRAM device by a high-resolution transmission microscopy (HR-TEM), by which GeO_*x*_ switching layer with 3-nm thickness is confirmed. The schematic of the fabricated ReRAM cells is shown in Fig. 1b. Ni and *p*^+^ Si act as the materials for the top electrode (TE) and bottom one (BE), respectively. Figure 2a shows the measured *I*–*V* curves from the fabricated ReRAM device with a diameter of 100 μm after 1, 5, 10, and 20 direct-current (DC) sweeps using a Keithley 4200A, with 0.1-mA compliance current. The distributions of set and reset voltages are confirmed to be narrow owing to the nonmetallic switching dielectric material formed by a MTO and finalized by a post-deposition annealing (PDA). In order to elucidate the conduction mechanism in the Ni/GeO_*x*_/*p*^+^-Si ReRAM cell, *I*–*V* curves in the high-resistance state (HRS) in the positive voltage HRS region and in the negative voltage LRS region are depicted in Fig. 2b and inset. The conduction mechanisms in the three regions of HRS state can be categorized into space-charge-limited current (SCLC) mechanism which follows the relation *I* ∝ *V*^*α*^. In Fig. 2b, different slopes are obtained depending on regions: region I (voltage < 0.7 V) with a slope of 1.83, region II (0.7 V ≤ voltage < 2.5 V) with a slope of 2.49, and region III (voltage > 2.5 V) with a slope of 4.78. In many previous reports, it was proven that different slopes could be extracted even under a single mechanism of SCLC [26–31]. Ohmic conduction follows the *I–V* relation with *α* ~ 1, dependency with Child’s square law can manifest with *α* ~ 2, and trap-filled conduction has the predominance with *α* > 2. Although linear relation has not been found in the HRS of the fabricated device, the other regions are governed by SCLC with different powers. In the region of relatively low voltage in HRS, the trap centers inside the GeO_*x*_ layer are filled by the weak carrier injection from the Ni TE. Carrier transport in this region is effectively described by the Child’s law where the current density (*J*) is expressed in a closed form [32–37]:

1 $$J_{{{\text{Child}}}} = \frac{9}{8}\kappa \mu \frac{{V^{2} }}{{t^{3} }}$$

**Fig. 3:**
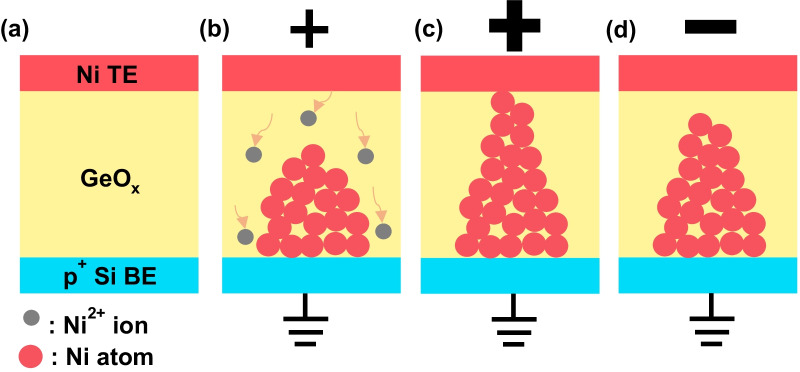
Schematic of the switching process of the Ni/GeO_*x*_/p^+^-Si ReRAM device (larger sign denotes larger bias). **a** Pristine state. **b** Ni^2+^ ions (gray circles) move toward the *p*^+^ Si bottom electrode and undergoes reduction to form Ni atoms (red circles), by which a conductive filament is formed and grows toward the Ni top electrode. **c** Conductive filament touches the top electrode (LRS). **d** Rupture of the conductive filament due to the application of opposite polarity bias voltage (HRS)

**Fig. 4:**
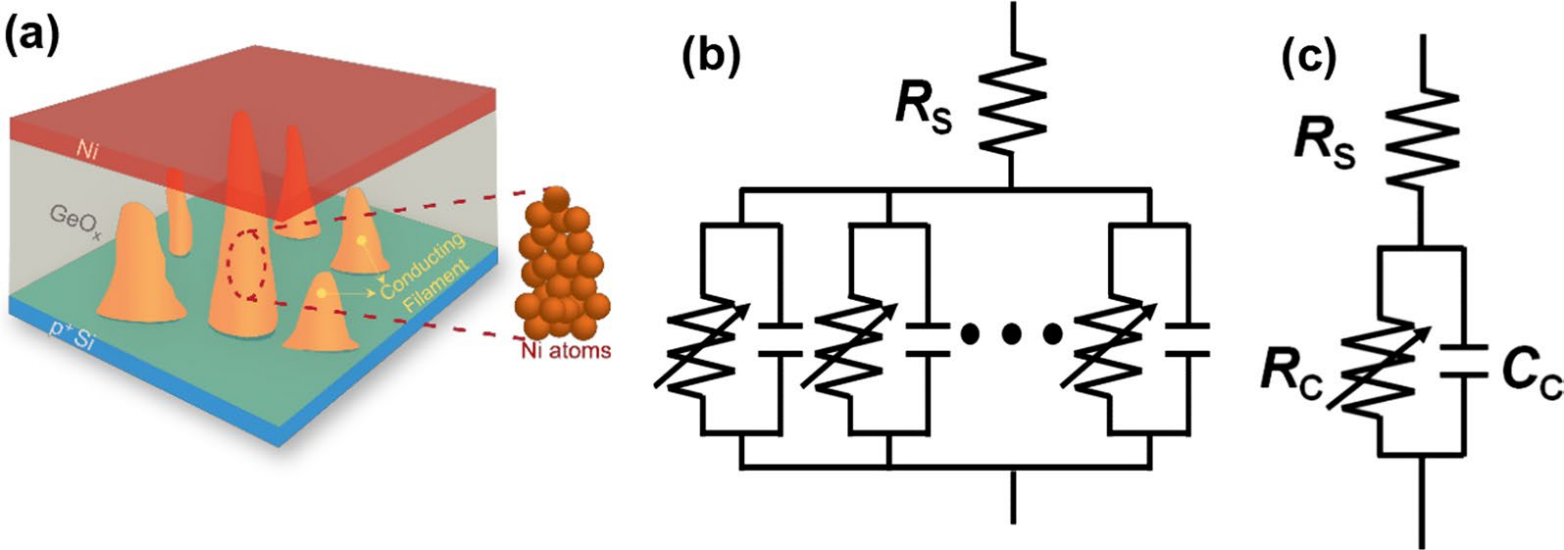
Physical and electrical representations of a GeO_*x*_ ReRAM cell. **a** Three-dimensional schematic of an ReRAM cell and the conducting filaments. **b** Electrical circuit model considering the multiple growths of conducting filaments. **c** Simplest ReRAM equivalent circuit

**Fig. 5:**
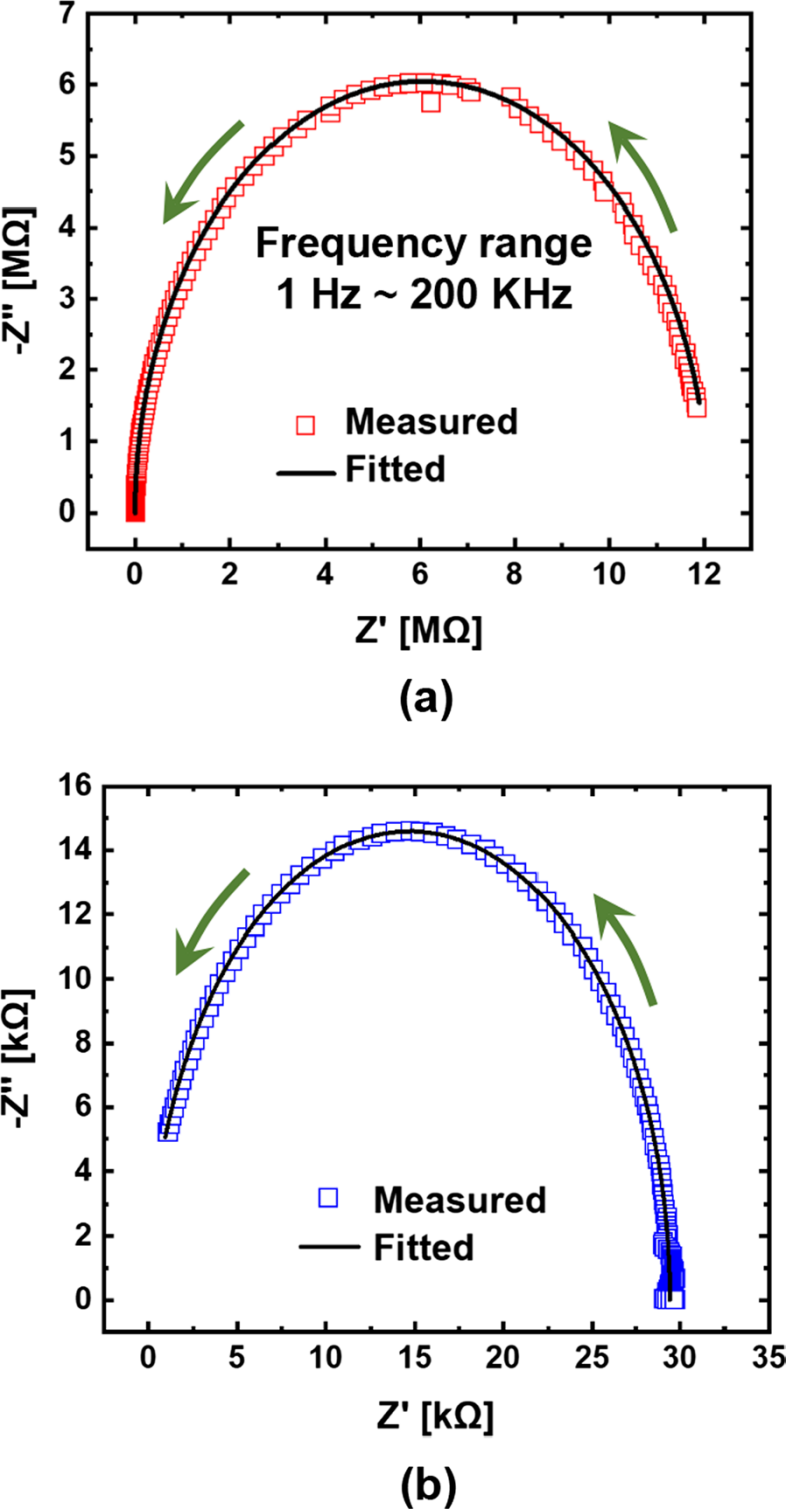
Impedance analysis of the fabricated GeO_*x*_ ReRAM device in the complex plane by a chemical impedance analyzer. Cole–Cole plots of the device in the **a** HRS at TE voltage = 2.3 V and **b** LRS at TE voltage = 0.7 V. The arrows in the figures indicate the directions of frequency sweep (counterclockwise directions) during the impedance analyses

Here, *κ* is the dielectric constant of GeO_*x*_, *μ* is the carrier mobility across the dielectric, *V* is the applied voltage, and *t* is the thickness of GeO_*x*_ layer. This region is also known as the trap-mediated SCLC region [38, 39]. As the voltage increases, the injected carriers begin to have the predominance over the thermally generated ones in number within the switching layer (region II) and the slope further increases. As the voltage goes very high, strong carrier injection takes place and all the trap states inside the switching layer are occupied by the carriers. In this region (region III), the conduction is made without being affected by traps and becomes completely space-charge-dependent, by which it is called trap-filled SCLC [40]. In case of LRS state in the negative TE voltage region, the slope is extracted to be 1.06 as shown in the inset of Fig. 2b, in which the current conduction mechanism can be mainly explained by ohmic conduction. The endurance and retention characteristics of the GeO_*x*_ ReRAM devices are demonstrated in Fig. 2c, d, respectively. The enhanced device reliability is evident from the high on/off ratio reaching 4.8 × 10^3^ and the retention time longer than 10^4^ s as shown in Fig. 2c, d, which reveals an explicit improvement in comparison with a device having the similar configuration reported in the previous literature [21]. Figure 3a–d illustrates the construction and destruction of the conducting bridge in the Ni/GeO_*x*_/*p*^+^-Si ReRAM device. There is no conduction filament in the pristine state (Fig. 3a), but Ni^2+^ ions begin to penetrate into the GeO_*x*_ switching layer as the TE voltage increases. These Ni^2+^ ions are reduced at the BE resulting in the gradual growth of conductive filaments of Ni atoms toward the TE (Fig. 3b). As the TE voltage increases, the filament formed by the Ni atoms touches the TE and the resistance state turns to LRS (Fig. 3c). As the TE voltage is reduced and goes into the negative region, the conductive filament undergoes electrochemical dissolution and gets ruptured leading to the HRS state (Fig. 3d). This formation and rupture of the conducting filament, or conducting bridge, are realized by the metallic species, which is more likely to be observed in the ReRAM cells employing Ni as the TE material [41, 42]. Oxygen vacancy migration has been identified as a dominating mechanism for formation of conductive filament in the oxide-based ReRAMs. While the possibility of oxygen vacancy formation still remains in the operation principle of the fabricated device, the essential point that needs to be conveyed lies in the fact that the predominance might have been moved to formation of metallic conductive filament based on Ni atoms in conduction mechanism. This is due to the electrochemically active nature of Ni which can easily form metallic conductive filaments inside the oxide dielectric, which also can be supported by previous studies carried out by Sun et al. [42]. In addition, a main requirement for stable and reliable oxygen migration is the formation of oxygen reservoir layer (ORL) typically formed close to the metallic anode [43]. There is no explicit presence of ORL in the fabricated device as can be confirmed by the TEM image in Fig. 1a, since the deposited Ge was thermally oxidized in the O_2_ ambient at 550 °C and further densified over the additional annealing at 600 °C, which suppressed the chances to form a mixed-phase layer, ORL, between the Ni anode and the lower GeO_2_ layer [23]. The highest temperature over the device fabrication was 600 °C as mentioned above, and thus, we can put the entire process integration after front-end-of-the-line (FEOL) of the Si complementary metal–oxide–semiconductor (CMOS) integrated circuits. The standard temperature for alloy with H_2_/N_2_ mixture in the CMOS processing that comes at the final step is usually around 450 °C. Thus, there is much room to insert the entire process integration of GeO_*x*_ ReRAM in the back-end-of-the-line (BEOL) of Si CMOS processing. Considering the fact that there are many candidates for Si processing-compatible metals with melting points higher than 600 °C for constructing gate, barrier, and interconnect, including Ti, TiN, W, Cu, and poly-Si, the ReRAM arrays can be fabricated even after all the metallization in the Si CMOS integrated circuits are completed if proper electrical isolation is warranted by depositing inter-layer dielectrics (ILDs). The ReRAM array can be integrated with CMOS circuits either vertically or horizontally. If low-melting-point metals are required in some parts of interconnection, it can be realized by at the far-back-end-of-the-line at the same time not to distort the material properties, doping profiles, and critical dimensions defined in the previous stages. Monolithic 3D integration of ReRAM on Si CMOS circuits was reported with a processing temperature of 525 °C for 90 min [36]. Although the processing temperature for oxidation and PDA were 550 °C and 600 °C, respectively, the processing times were much shorter, for 10 min and 20 min, respectively. Thus, it should not be a threat to the Si processing compatibility in terms of not only material but also thermal budget. The annealing can be further adjusted with lower temperature and prolonged time [23]. The conducting filaments repeating the construction and destruction with voltage dependence shown in Fig. 3a–d are randomly distributed over the ReRAM cell as illustrated in Fig. 4a. Each filament can be described as a parallel combination of a voltage-dependent resistance and a capacitance as shown in Fig. 4b. The series resistance (*R*_s_) at the top of the block comes from the series combination of TE, BE, and contact resistances. Since all the filaments are connected in parallel between TE and BE, all the resistances can be lumped into an equivalent cell resistance (*R*_c_), and likewise, all the parallel capacitances are summed into an equivalent cell capacitance (*C*_c_) as demonstrated in Fig. 4c. Although the construction and destruction of the conducting bridge are explained by the movements of the metallic atoms and the bridging mechanism can be varied according to the material combination making up the cell stack, an individual cell can be described by a variable resistor and a capacitor, and thus, the suggested equivalent electrical circuit model in Fig. 4b, c is allowed to have the high universality for ReRAM devices. In order to extract the passive elements in the ReRAM cell, the fabricated devices were brought to an impedance analyzer, IM3590 by Hioki, with introducing the equivalent circuit model in Fig. 4c. The Cole–Cole plots from the fabricated ReRAM device in the HRS and LRS are shown in Fig. 5a, b, respectively. The measurement frequency was varied from 1 Hz to 200 kHz, and the *x* (*Z*′) and *y* (*Z*″) axes indicate the real and imaginary parts of the impedance. We applied different voltages for extracting the impedances at HRS and LRS. A high DC bias can be desirable for obtaining explicit capacitance values. However, in order for preserving the switching layer quality relatively more vulnerable in the LRS state and device reliability over the long-time frequency sweep period under a DC bias stress, the value was lowered to 0.7 V for the measurement in the LRS. Once the bias voltage is lower than the set voltage (~ 3.8 V in this work), it was experimentally confirmed that there was no significant change in impedance analysis results with a change in bias voltage in performing the frequency sweep [44]. As the frequency goes higher, the trajectory is plotted in the counterclockwise direction. The appearance of a single semicircle in the Cole–Cole plot is an affirmation of the fact that the charge transport mechanism in the device can be described in terms of a parallel *RC* circuit as described in Fig. 4c. The square symbols in Fig. 5a, b show the measurement results whereas the continuous lines denote the fitted data. Table 1 shows the values of the extracted parameters from the impedance analysis of the GeO_*x*_ ReRAM device. It is revealed that the capacitance in the LRS is much smaller than that in the HRS, which attributes to the reduction in effective area for the device capacitance taking place over the growth of a conductive filament. The higher accuracy in the impedance analysis fitting shows that the physical simplification of a realistic ReRAM cell in Fig. 4a and the equivalent circuits in Fig. 4b, c induced from the results in Fig. 4a have high coherence.

**Table 1:** Values of passive elements extracted from the impedance analyses

Resistance states	*R*s (Ω)	*R*c (MΩ)	*C* (pF)	Extraction voltage (V)
HRS	197	12.1	163	2.3
LRS	216	29.2	116	0.7

### Training Approach and Hardware Architecture of the PIM with GeO_*x*_ ReRAM

**Fig. 6:**
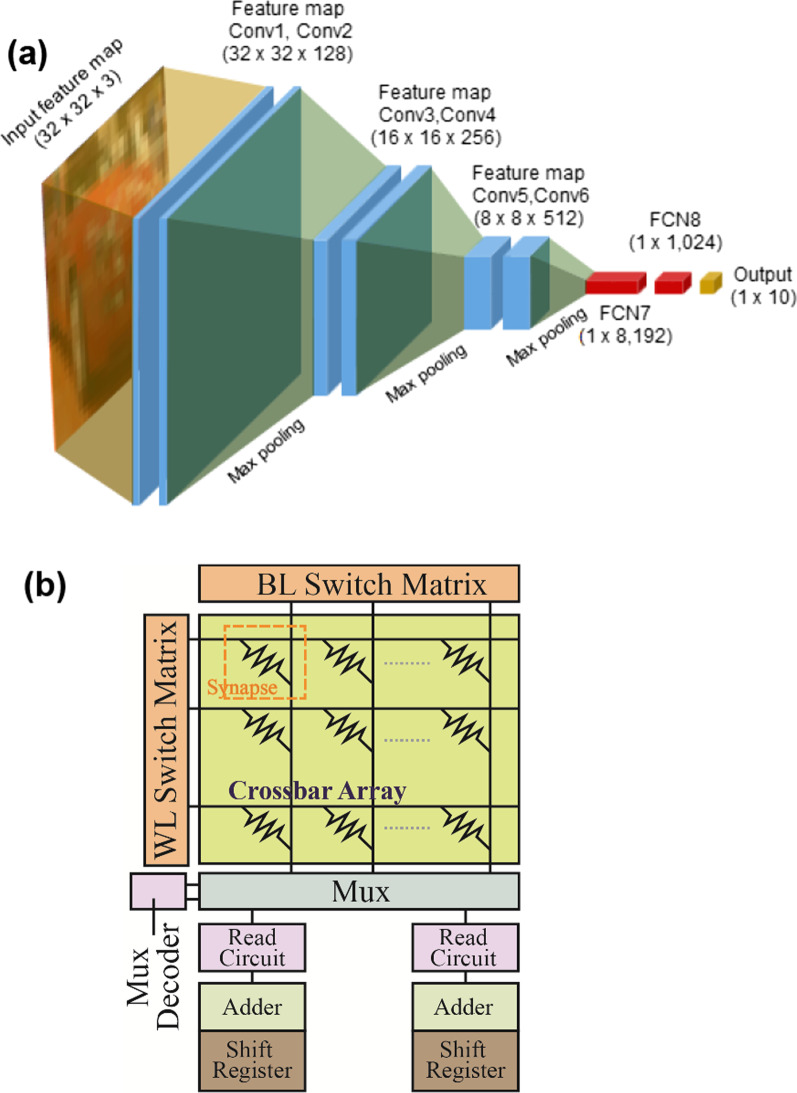
Schematic of neural network and PIM architecture. **a** VGG-8 neural network for CIFAR-10 image recognition. The convolutional layers are marked as Conv1 to Conv6, and the corresponding feature maps are indicated. The fully connected (FCN) layers are identified as FCN7 and FCN8 with their dimension of weights in the brackets. **b** Design of a tile of PIM architecture embedding the GeO_*x*_ ReRAM synapse array

The off-chip training capability for graphical image recognition by the GeO_*x*_ ReRAM has been evaluated using the Canadian Institute for Advanced Research (CIFAR)-10 dataset in the Visual Geometry Group (VGG)-8 neural network architecture. The architecture of the VGG-8 network comprises a total 8 layers: 6 convolutional layers and 2 fully connected layers. The detailed schematic of the VGG-8 network architecture is shown in Fig. 6a. The input CIFAR-10 dataset has a collection of 60,000 color (red–green–blue) images of 32 × 32 resolution. The images can be broadly classified into 10 output indexes. During the network training, the data is grouped into 50,000 train and 10,000 test images with a batch size of 200. The VGG-8 network has been trained using a stochastic gradient descent (SGD) algorithm and rectified linear unit (ReLU) activation function. The realization of hardware-sense neural network for a PIM architecture is illustrated in Fig. 6b [25]. The hardware design is capable of evaluating the performance of the VGG-8 network of GeO_*x*_ ReRAM synaptic devices. The system takes into account the various hardware constraints including technology node, analog-to-digital converter (ADC) precision and the nonideal changes in the synaptic weights during the training. The system design has been hierarchically organized into chip level, processing element level, and synaptic array level elements. For the full single-chip hardware integration, peripheral circuits including ADCs, buffers, multiplexers (MUX), interconnects with the 32-nm predictive technology SPICE model parameters have been presumably used and other relevant circuitry such as digital adders and shift registers have been also considered. The accumulation circuits include the chip-level units, processing element level adders, tile-level adders, and shift adders on the edges of the ReRAM synapse array. The system-level performance has been evaluated using an analog parallel read-out scheme using 64 × 64 synaptic array size and 5-bit ADC precision. The input data flow into the wordline (WL) switch matrix, and the MAC operations in the crossbar array generate partial sums which are accumulated along the columns using the read-out circuits (flash ADCs). The bit-quantized ADCs are much larger in area than the synaptic array column pitch and hence they share several columns using the column MUX. The roles of adders and shift registers are to shift and accumulate partial sums by the MAC operations over repeated cycles due to batch-wise data processing. A major concern with the batch-wise data processing lies in the large amount of intermediate data generated during the feed-forward process taking place in the computation of activations. In order to minimize the requirement for in-chip memory space, the PIM architecture can be designed to send the intermediate data to off-chip DRAM, which can be optional depending on neural network size and chip area of the GeO_*x*_ ReRAM PIM architecture.

### System-Level Performance Evaluation

**Fig. 7:**
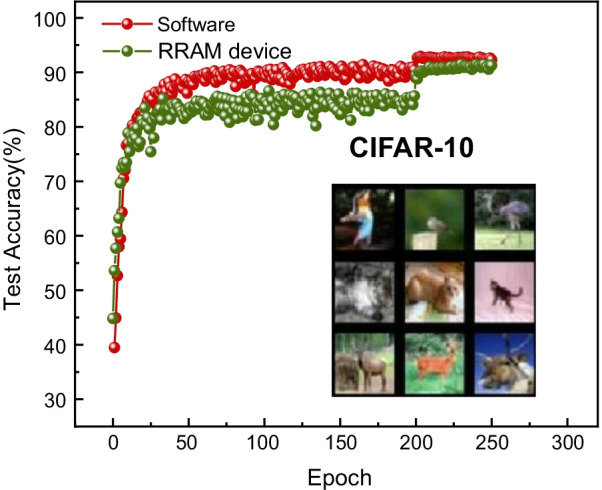
Accuracy in image test as a function of the number of training epochs for the GeO_*x*_ synapse array in comparison with the software neural network. The inset shows a subset of the CIFAR-10 input data

**Fig. 8:**
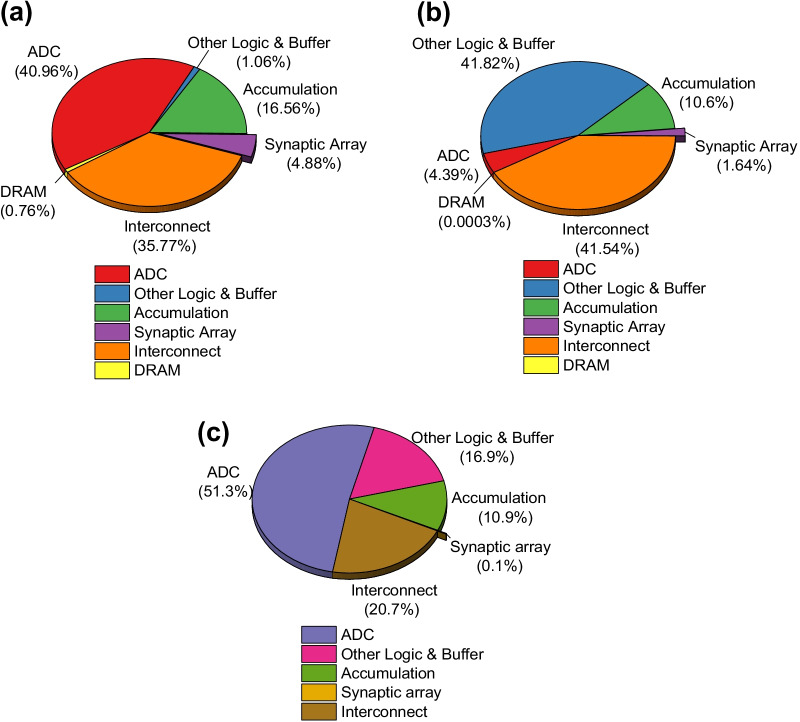
Pie charts illustrating the distributions of major metrics. **a** Energy consumption, **b** latency, and **c** area occupied by various hardware elements in the chip-level PIM architecture. Except the off-chip DRAM memory domain used for the storage of intermediately generated data from the PIM chip (can be optional depending on architecture design), all the other components can be presumably implemented on chip

For evaluating the system-level performances of the PIM architecture based on GeO_*x*_ ReRAM, binary-state switching operations in the synaptic array were assumed with potentiation (write) voltage = 3 V with a pulse width = 100 µs and inference voltage = 0.7 V with a pulse width = 100 µs. Figure 7 shows the accuracy in CIFAR-10 image recognition as a function of number of epochs in comparison between software and hardware neural networks. It is observed that the PIM system with the hardware neural network of GeO_*x*_ ReRAM synapses has achieved an accuracy of 91.27%, which is comparably high with the accuracy obtained by the software neural network, 92.31%, in terms of test accuracy. A sharp jump is witnessed in both the software and hardware-based trainings at 200 epochs. This is due to the decreasing learning rate strategy employed after 200 epochs for the optimized training of the network. Inset of Fig. 8 shows a subset of the CIFAR-10 dataset. The system-level parameters from the simulation of the designed PIM architecture are summarized in Table 2. Figure 8a–c shows the pie diagrams of portions in energy, latency, and area occupied by different hardware components in the PIM architecture. The energy distribution in Fig. 8a reveals that the ADCs (multi-level current sense amplifier) and interconnects consume the largest energy, followed by the accumulation circuits. The synaptic array energy consumption is extremely low as compared to other components. The latency distribution in Fig. 8b shows that the logic and buffer circuits along with the interconnects have the predominance in determining the overall system latency. The large size of the VGG-8 network results in the considerable amount of on-chip data transfer from the buffer memory and a large number of synapses in the array leading to increase complexity in interconnects within the PIM chip. This is a crucial factor in limiting the overall chip latency. Finally, it is observed from Fig. 8c that the total chip area is largely occupied by the ADCs. Thus, the ADC area needs to be intensively optimized with regard to both energy and area efficiencies. The energy, latency, and area minimally consumed by the GeO_*x*_ ReRAM synapse array are an indication of its high applicability in the hardware PIM architecture. Further, the computational demands of the VGG-8 network on the hardware PIM design are also evaluated in order for understanding the future directions for the optimization of hardware neural network architecture. In order to calculate the energy distribution across the VGG-8 layers, firstly, the simulator maps the trained synaptic weights in each layer (per epoch) into a hierarchical organization of tiles, processing elements (PEs), down to synaptic arrays. This weight mapping is proceeded in the manner that the memory domain inside the chip is optimally utilized. Once the weight mapping is completed, the inference operations are carried out for each epoch using the images from the test dataset in the CIFAR-10 dataset. During the inference, the energy distribution of each layer is calculated as the sum of energies consumed by the synaptic array and subcircuit modules such as analog-to-digital converter (ADC), switch matrix, multiplexer, etc. For the energy calculation in the subcircuit module, the dynamic energy consumption at each node is calculated to be *CV*_DD_^2^ by the effective total capacitance across a logic gate (*C*) and drive voltage (*V*_DD_) and summed up to obtain the total energy consumption in the subcircuit module for a single operation. This is further multiplied by the number of operations in the subcircuit module to calculate the total dynamic energy consumed by a specific module. For calculating the energy consumption in the synapse array, two components are considered: static energy consumed by a synapse and dynamic energy consumed by the parasitic capacitance inside the array. The static energy consumption of a synaptic device is obtained by the conductance of an ReRAM (*G*) (synaptic weight), inference voltage (*V*_inf_), and inference pulse width (*T*_inf_) as follows:

2 $${\text{Energy}}_{{{\text{synapse}}}} = {\text{GV}}_{{{\text{inf}}}}^{2} T_{{{\text{inf}}}}$$

**Table 2:** Chip-level parameters and performances computed per epoch for the GeO_*x*_ ReRAM synapse array-based PIM architecture

PIM chip parameters	Values
Chip area	62.5 mm^2^
Total energy on chip	3.35 × 10^–5^ J
Latency	1.33 ms
Peak energy efficiency	58.92 TOPS/W
Mean energy efficiency	36.42 TOPS/W
Inference energy in the synapse array	1.64 × 10^–6^ J
Other logic energy	3.55 × 10^–7^ J
ADC energy	1.37 × 10^–5^ J
Interconnect energy	1.20 × 10^–5^ J
Inference latency in the synapse array	2.20 × 10^–5^ s
Other logic latency	5.58 × 10^–4^ s
ADC latency	5.86 × 10^–5^ s
Interconnect latency	5.55 × 10^–4^ s

**Fig. 9:**
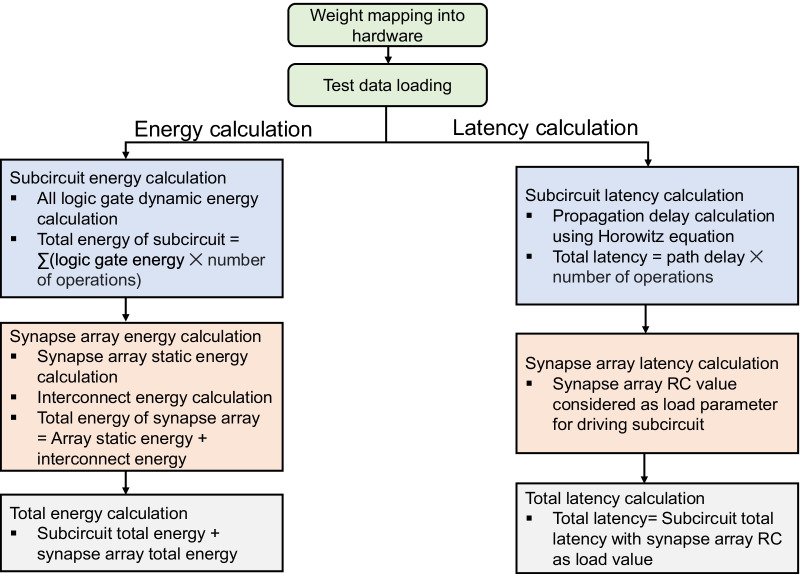
Workflow depicting the sequential steps involved with calculations of energy and latency over the layers in the VGG-8 neural network

**Fig. 10:**
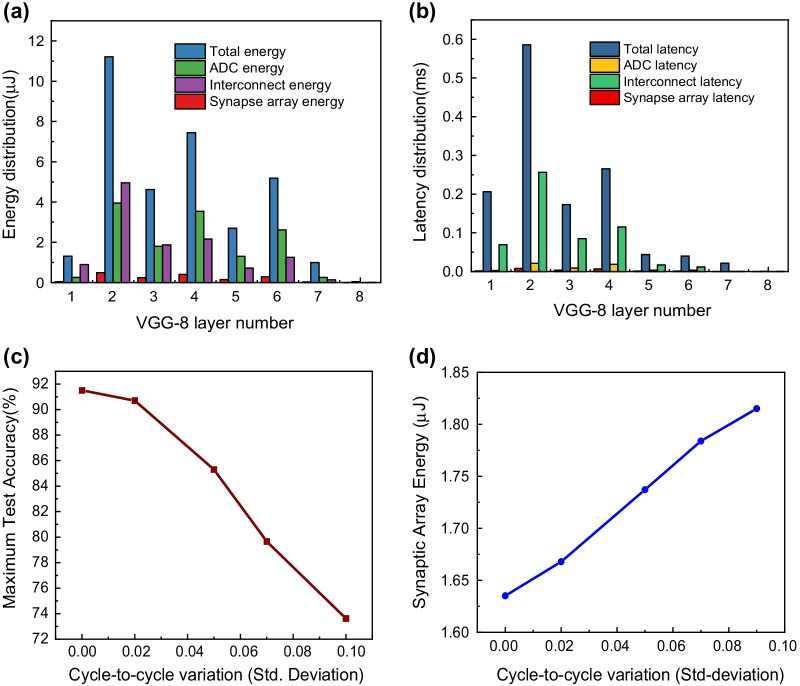
Histograms demonstrating **a** energy and **b** latency distributions across the different computational layers in the VGG-8 network. **c** Maximum test accuracy and **d** inference energy in the synapse array as a function of variation in the cycle-to-cycle GeO_*x*_ ReRAM operations

The dynamic energy consumption in the array interconnect is calculated based on *RC* analysis. In the similar manner, the latency distributions across individual layers are obtained as the sum of latency of the subcircuit modules (multiplied by the number of operations) with the *RC* values in the synapse array as the load. The Horowitz’ equation has been used for calculating the latency of logic gates in the subcircuits [45]. The latency over the synapse array is accommodated into the total latency by considering the synapse array *RC* values as the load parameters for the subcircuit modules connected to the synapse array. Further details about the architectural and performance estimation details of the system-level simulations can be explained [25]. Figure 9 depicts the flowchart which gives the detailed description of the procedure for calculation of energy distribution and latency distribution across the VGG-8 layers. The as-computed layer-wise energy consumption and latency distributions of the VGG-8 network are shown in Fig. 10a, b. It is observed that the convolutional layers with additional pooling layers (previously shown in Fig. 6a), i.e., layers 2 and 4, demand the largest energy and time consumptions for the in-memory computations. Judging from both the histograms, it is clarified that the inference energy and latency of the synaptic array across all the layers of VGG-8 are minimized by the virtues of the fabricated GeO_*x*_ ReRAM. However, it is important to consider the variation in the device-level operations in evaluating the system-level performances. Recently, there have been several studies on the effects of nonideal variations in the synaptic devices on the PIM system performances [46–48]. As one of the most decisive nonidealities, variation in cycle-to-cycle switching operations can be quantified as a standard deviation and can be treated as an independent variable in determining the system accuracy. Figure 10c shows the maximum test accuracy for the CIFAR-10 image recognition as a function of the cycle-to-cycle variation. It is explicitly shown that there is little drop in the accuracy up to the standard deviation of 0.02, which confirms the robustness of the GeO_*x*_ ReRAM synaptic devices implementing the PIM architecture. It is demonstrated in Fig. 10d that the inference energy monotonically increases with the standard deviation but the system preserves the robustness against the device-level variation up to standard deviation of 0.02.

## Conclusion

ReRAM cells featuring the Ni/GeO_*x*_/*p*^+^-Si stack with a high Si processing compatibility have been fabricated and characterized, with a particular interest in implementing highly-scalable nonvolatile memory-based PIM architecture. The fabricated ReRAM device has demonstrated increased reliability due to the medium-temperature oxidation process. The circuit and performance parameters of the fabricated GeO_*x*_ ReRAM were fed into the system-level simulation with realistic peripheral circuitry to evaluate the system accuracy in image learning and the applicability of the GeO_*x*_ ReRAM technology for the future computing architecture. The CIFAR-10 image recognition accuracy and hardware parameters have been evaluated in consideration of device-level nonideality. The energy consumption, latency, and area occupied by the synaptic array are observed to be the smallest in comparison with other functional modules in the PIM architecture. The computational demands of the pooling layer in the VGG-8 network on the overall chip energy consumption has been revealed by layer-wise neural network evaluation. In conclusion, a high image recognition accuracy above 90%, high energy efficiency, low latency, and minimal area requirement warrant that the GeO_*x*_ ReRAM can be a plausible candidate for realizing the chip-packaged PIM architecture.

## Methods

### Device Fabrication

The proposed ReRAM devices were fabricated in the class-controlled Si nanofabrication facility. After preparing and initial cleaning of 6″ *p*-type (100) Si wafers, ion implantation was performed with a dose of B 5 × 10^15^ cm^−2^ at an acceleration energy of 40 keV. Dopant activation was carried out in the furnace at 900 °C for 20 min, and Ge of 3 nm thickness was deposited by a thermal evaporator at 96-A source current and 40 × 10^–6^ torr vacuum pressure. Then, dry oxidation of Ge was performed by a medium-temperature oxidation (MTO) at 550 °C with an O_2_ flow of 7,250 sccm for 10 min. and the wafers were sent to a thermal tube for an additional annealing at 600 °C with an N_2_ flow of 5000 sccm for 20 min. Lithography was performed using a mask aligner for circular patterns. 200-nm-thick Ni was deposited on the GeO_*x*_ switching layer, and then, acetone and isopropyl alcohol (IPA) were put in the cyclic uses for lift-off and residual removal processes. Finally, the wafers were rinsed in the de-ionized (DI) water and completely dried for finishing the device fabrication.

### Electrical Measurement

Electric switching characteristics of the fabricated GeO_*x*_ RRAM device were obtained at room temperature using a Keithley 4200A-SCS semiconductor parameter analyzer inside an electrically shielded probe station. The impedance analyses were carried out using a Hioki IM3590 impedance analyzer in the air ambient.

### System-Level PIM Evaluation Environments

The system-level simulations were carried in a high-end workstation employing a 32-core AMD Ryzen 9 Processor as the central processing unit (CPU) and an NVIDIA RTX 3090 as the graphic processing unit (GPU). For the neural network training, a stochastic gradient descent (SGD) algorithm was adopted with rectified linear unit (ReLU) activation function. During the network training, a batch size of 200 and a learning rate of 1 were used. The weight and the gradient were considered to have 5-bit precisions, whereas the activation and the error were computed with 8-bit precision.

## Data Availability

Not applicable.
